# Hippocampal Structures Among Japanese Adolescents Before and After the COVID-19 Pandemic

**DOI:** 10.1001/jamanetworkopen.2023.55292

**Published:** 2024-02-08

**Authors:** Lin Cai, Norihide Maikusa, Yinghan Zhu, Atsushi Nishida, Shuntaro Ando, Naohiro Okada, Kiyoto Kasai, Yuko Nakamura, Shinsuke Koike

**Affiliations:** 1 Center for Evolutionary Cognitive Sciences, Graduate School of Arts and Sciences, The University of Tokyo, Tokyo, Japan; 2 Research Center for Social Science and Medicine, Tokyo Metropolitan Institute of Medical Science, Tokyo, Japan; 3 Department of Neuropsychiatry, Graduate School of Medicine, The University of Tokyo, Tokyo, Japan; 4 The International Research Center for Neurointelligence, University of Tokyo Institutes for Advanced Study, Tokyo, Japan; 5 University of Tokyo Institute for Diversity and Adaptation of Human Mind, The University of Tokyo, Tokyo, Japan

## Abstract

**Importance:**

Few studies have used a large-sample, longitudinal, population-based cohort study to examine whether the COVID-19 pandemic as a global major life event is associated with structural plasticity of the adolescent hippocampus.

**Objective:**

To examine whether Japan's first state of emergency (SoE) during the COVID-19 pandemic was associated with alterations in the macrostructures and microstructures of the hippocampus during its development.

**Design, Setting, and Participants:**

The population-neuroscience Tokyo TEEN Cohort study is a prospective cohort study with 4 consecutive waves in Tokyo, Japan. Due to the SoE, data collection was suspended between March 27, 2020, and July 30, 2020. Analyzed data, comprising 1149 brain structural scans obtained from 479 participants, of whom 336 participants had undergone 2 or more scans, were collected between October 2013 and November 2021. Data were analyzed from August 2022 to December 2023.

**Exposures:**

Japan’s first SoE (April 7 to May 25, 2020).

**Main Outcomes and Measures:**

Hippocampal volume, 12 hippocampal subfield volumes, and 7 microstructural measures of the hippocampus.

**Results:**

A total of 1060 brain scans from 459 participants (214 female participants [47%]) including 246 participants from wave 1 (median [IQR] age, 11.3 [11.1-11.7] years), 358 from wave 2 (median [IQR] age, 13.8 [13.3-14.5] years), 304 from wave 3 (median [IQR] age, 15.9 [15.4-16.5] years), and 152 from wave 4 (median [IQR] age, 17.9 [17.5-18.4] years) were included in the final main analysis. The generalized additive mixed model showed a significant associations of the SoE with the mean hippocampal volume (β = 102.19; 95% CI, 0.61-203.77; *P* = .049). The generalized linear mixed models showed the main associations of the SoE with hippocampal subfield volume (granule cell and molecular layer of the dentate gyrus: β = 18.19; 95% CI, 2.97-33.41; uncorrected *P* = .02; CA4: β = 12.75; 95% CI, 0.38-25.12; uncorrected *P* = .04; hippocampus-amygdala transition area: β = 5.67; 95% CI, 1.18-10.17; uncorrected *P* = .01), and fractional anisotropy (β = 0.03; 95% CI, 0.00-0.06; uncorrected *P* = .04).

**Conclusions and Relevance:**

After the first SoE, a volumetric increase in the hippocampus and trend increase in 3 subfield volumes and microstructural integration of the hippocampus were observed, suggesting that the transient plasticity of the adolescent hippocampus was affected by a major life event along with the typical developmental trajectory.

## Key Points

**Question:**

Was the COVID-19 pandemic associated with alterations in the hippocampal structure in adolescents?

**Findings:**

In this cohort study of 479 adolescents living in Tokyo that included 1060 structural scans, a volumetric increase in the hippocampus and some of its subfields, as well as an increased hippocampal microstructural integration in the course of adolescent development, were found after the COVID-19 pandemic.

**Meaning:**

These findings suggest that Japan’s first state of emergency declared for the COVID-19 pandemic was associated with changes in the hippocampal macrostructures and microstructures with implications for hippocampal plasticity and future pandemic preparedness.

## Teaser

This cohort study evaluates whether Japan's first COVID-19 state of emergency was associated with alterations to the macrostructures and microstructures of the hippocampus among adolescents in Tokyo.

## Introduction

Although a severe acute respiratory coronavirus 2 (SARS-CoV-2) infection has been revealed to result in changes in the structure and function of the human brain,^1,2,3^ the association between the COVID-19 pandemic and the brain among uninfected individuals is still underexplored. Since the declaration of the COVID-19 pandemic in March 2020, many countries have effectively reduced infection rates by implementing lockdowns. However, lockdowns and related measures had a tremendous impact on the mental health of the general population, especially children and adolescents.^4,5^ Since adolescence is a sensitive period for the development of mental illnesses caused by stressful life events,^6,7^ it is important to understand the effect of stressful experiences on the adolescent brain,^7,8,9^ particularly the hippocampus with its high plasticity and susceptibility to stressful experiences.^10^ However, the mechanism by which stressful experiences affect the human adolescent hippocampus is difficult to investigate because it is not permitted to manipulate stress for humans in an experimental setting. Thus, the COVID-19 pandemic, as an extremely stressful life event, enabled us to examine the associations of stress with the adolescent hippocampus.

Only a few preliminary studies have suggested that compared with adolescents scanned before the COVID-19 pandemic, adolescents during the COVID-19 pandemic showed increased volume in the hippocampus, which may reflect accelerated brain maturation due to the COVID-19 pandemic.^11,12^ However, extant studies still have some limitations, such as (1) the participants were measured only before and after the COVID-19 pandemic and were not tested in a prospective design according to adolescent development, (2) the participants were not recruited based on population representativeness, and (3) the studies only focused on the macrostructural alterations in the hippocampus, ignoring the alterations in hippocampal subfields and microstructure.

**Figure 1.**
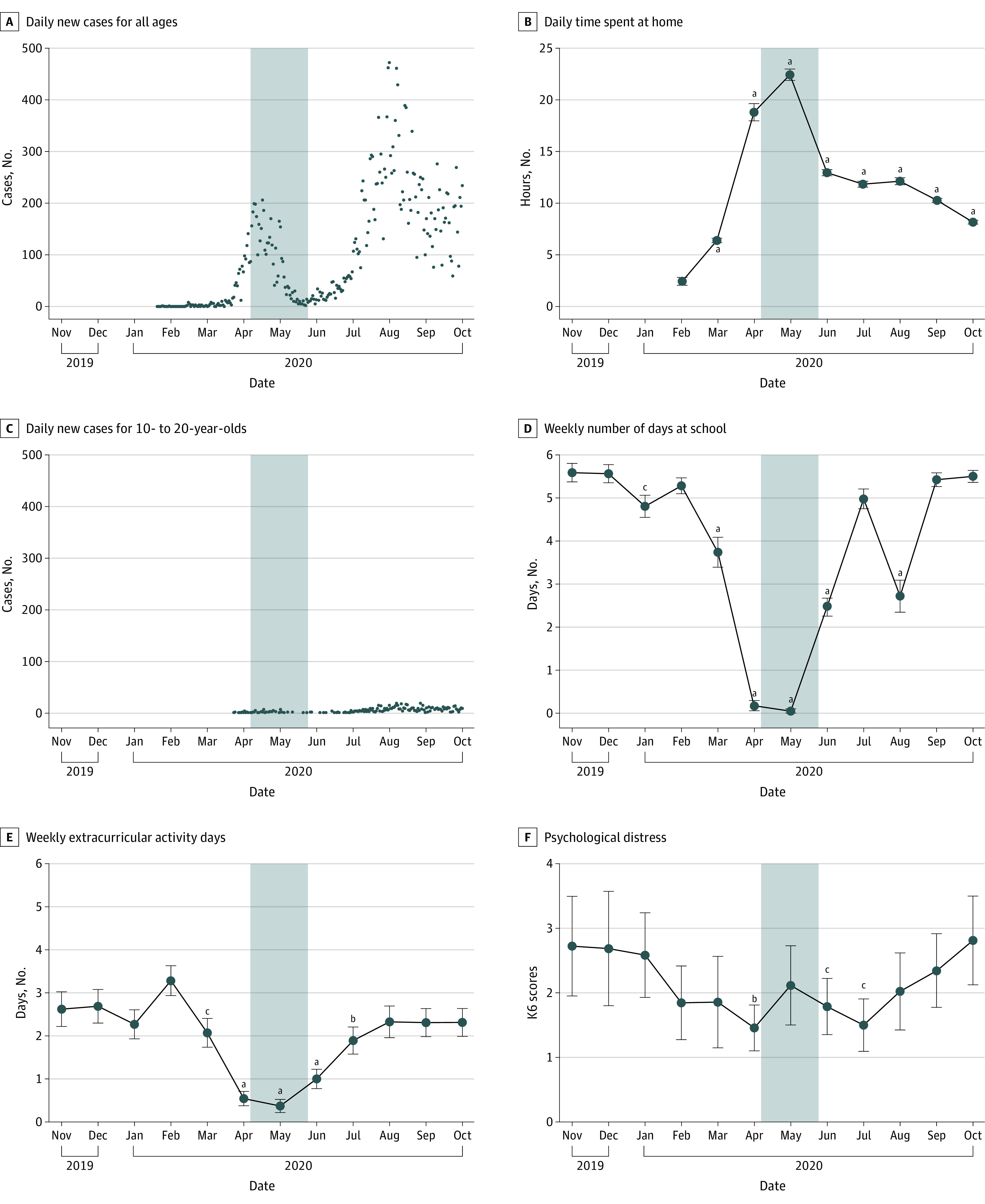
COVID-19 Cases and Mobility of Citizens in Tokyo From November 2019 to October 2020 The period of Japan’s first state of emergency (April 7 to May 25, 2020) is indicated by the blue shaded areas. Daily number of newly reported cases of COVID-19 is plotted for all ages (A) and individuals aged 10 to 20 years in Tokyo (C), using data from the Ministry of Health, Labour and Welfare in Japan. Daily time spent at home per month is plotted for citizens in Tokyo using Google COVID-19 Community Mobility Reports (B). The weekly number of days at school (D), weekly extracurricular activity days (E), and psychological distress (F) per month are plotted for general adolescents from pn-TTC subsamples. B, Reference month was February 2020. D to F, Reference month was November 2019. Relevant statistical methods and results are provided in eMethods 1 and eAppendix 1 in Supplement 1. ^a^*P* < .001. ^b^*P* < .01. ^c^*P* < .05.

On March 2, 2020, the Japanese government requested elementary, junior high, and high schools nationwide to give students temporary leave after spring break. Subsequently, the Japanese government declared the first state of emergency (SoE) on April 7, 2020, which requested residents refrain from leaving their homes for nonessential reasons and restricted the use of stores and facilities, yet without penalty for disobedience. This led to temporary closure of most Japanese schools until the end of May. After lifting the first SoE for the whole country on May 25, 2020, the Japanese government still instructed schools to adopt the measures of staggered attendance to avoid infections. The noncoercive restrictions of the first SoE effectively reduced the new daily reported cases (Figure 1A) and led to an increase in citizens’ daily time spent at home (Figure 1B) in Tokyo. Notably, the number of new daily reported cases for individuals aged 10 to 20 years was extremely low (Figure 1C).

To examine the associations of the COVID-19 pandemic with the hippocampal macrostructures and microstructures in adolescents, we analyzed data from the population-neuroscience Tokyo TEEN Cohort (pn-TTC) study project, which is a large-sample, longitudinal, population-based cohort study conducted in Tokyo, Japan,^13^ started in October 2013, that collected 1149 longitudinal structural brain scans from 479 participants as of November 2021. At the end of the third wave, due to the first SoE, data collection was suspended between the end of March 2020 and July 30, 2020. Given the COVID-19 pandemic may alter hippocampal structures by changing stress levels^8,9^ and/or environmental complexity^14^ among adolescents, we performed a preliminary analysis (eMethods 1 and eAppendix 1 in Supplement 1) using behavioral measurements from a subset of participants (65 participants) in the pn-TTC. We found that adolescents in this pn-TTC subset seldom went to school (Figure 1D) or attended extracurricular activities (Figure 1E) during the first SoE. Contrary to expectations, a significant decrease in psychological distress was observed among adolescents (Figure 1F). Despite experiencing disruptions in their daily routines due to the COVID-19 pandemic-related restrictions, adolescents in the pn-TTC could still encounter enriched environmental stimuli through increased usage of social media and virtual learning platforms, engaging in more online social interactions. Hence, we speculated that environmental complexity associated with hippocampal structural changes may remain stable. Contrastingly, stress reduction in adolescents in the pn-TTC, stemming from various factors such as increased time with family members and diminished pressure from examinations and peers, may be the primary cause of hippocampal changes.

As the first months of the pandemic are believed to have the most significant impact on the developing brain,^12^ we examined how the data collection time obtained through a logarithmic transformation was associated with hippocampal macrostructures and microstructures after the SoE. Accordingly, the primary hypothesis of the current study was that the SoE would cause an increase in the whole hippocampal volume during its development. Compared with the whole hippocampus, changes in hippocampal subfields and microstructure may occur more easily.^15,16^ Thus, the secondary hypothesis was that hippocampal subfields and microstructure would have similar changes due to the SoE.

## Methods

This cohort study was approved by the research ethics committees of the Faculty of Medicine, the University of Tokyo, Tokyo Metropolitan Institute of Medical Science, and the Graduate University for Advanced Studies. Written informed consent was obtained from the participants’ main caregivers and participants aged 15 years or older before participation. We followed the Strengthening the Reporting of Observational Studies in Epidemiology (STROBE) reporting guideline for observational studies.

### Participants

The pn-TTC project is an extension of the TTC study.^17^ The first wave of pn-TTC recruitment began in September 2013 and ended in February 2016. Subsequent waves 2 to 4 involved biennial participant recruitment. Data collection was conducted between October 2013 and November 2021. The participant eligibility criteria are provided in eMethods 2 in Supplement 1. As all participants in this study are from TTC wave 1, parental education level was obtained by a 6-point scale of educational attainment when their children were aged approximately 10 years. We used greater educational attainment of the father and mother as the socioeconomic status (SES) of the children.^18^ Simultaneously, participants’ intelligence quotient (IQ) at age 10 was assessed using a short version of the Wechsler Intelligence Scale for Children-–Third Edition.^19^

### Image Acquisition and Processing

For the 4 waves of data collection, two 3-T scanners and 3 acquisition procedures were performed. Only T1-weighted images were acquired for procedures 1 and 2, while T1-weighted, T2-weighted, and diffusion images were acquired for procedure 3. The detailed scan parameters for all procedures are found in eMethods 3 in Supplement 1. When only T1-weighted images were available, the legacy style of the Human Connectome Project (HCP) pipeline was used for image preprocessing. If both T1-weighted and T2-weighted images were available, the standard style of the HCP pipeline was used for image preprocessing.^20^ Then, bilateral hippocampal volumes were extracted from the FreeSurfer’s aseg.stats file, which were further harmonized using the traveling subject harmonization method to diminish the procedural difference (eMethods 4 in Supplement 1). For procedure 3, 12 hippocampal subfield volumes (ie, cornu ammonis 1 [CA1], CA2/3, CA4, subiculum, the granule cell and molecular layer of the dentate gyrus [GC-ML-DG], hippocampus-amygdala transition area [HATA], hippocampal tail, hippocampal fissure, molecular layer, presubiculum, parasubiculum, and fimbria) were obtained using the hippocampal subfield segmentation algorithm from T1-weighted and T2-weighted images in FreeSurfer version 6.0.0 (Laboratory for Computational Neuroimaging at the Athinoula A. Martinos Center for Biomedical Imaging).^21^ The raw diffusion scans were preprocessed using the HCP diffusion preprocessing pipeline.^20^ The microstructural metrics were estimated using the diffusion kurtosis imaging (DKI) model,^22^ as implemented in DIPY version 1.5.0 (DIPY Developers).^23^ The DKI model could produce 4 conventional diffusion tensor imaging images, ie, fractional anisotropy (FA), mean diffusivity, axial diffusivity, and radial diffusivity, and 3 kurtosis images, ie, mean kurtosis (MK), axial kurtosis (AK), and radial kurtosis (RK). For each scan, volumetric estimates for the bilateral hippocampi and hippocampal subfields and the 7 microstructural indices for the bilateral hippocampi were averaged across hemispheres as indices in the following statistical analysis. More details about the image processing are provided in eMethods 5 and 6 in Supplement 1.

### Statistical Analysis

All analyses were performed using R software version 4.2.2 (R Project for Statistical Computing). For exploring the adolescent developmental trajectory of the hippocampal volume, we built generalized additive mixed models (GAMMs) implemented with the mgcv package (version 1.8-42), including sex as a linear term, age as a smooth term using a penalized cubic regression spline and a basis function of 4, a tensor interaction term between age and sex, and intracranial volume (ICV) as a linear covariate (eMethods 7 in Supplement 1). For the developmental trajectory of hippocampal subfield volumes and DKI indices, generalized linear mixed models (GLMMs) were implemented with the lme4 package including the associations of sex and age, age × sex interaction, and ICV as a covariate (eMethods 7 in Supplement 1). All models included a random-effect intercept per participant.

**Figure 2.**
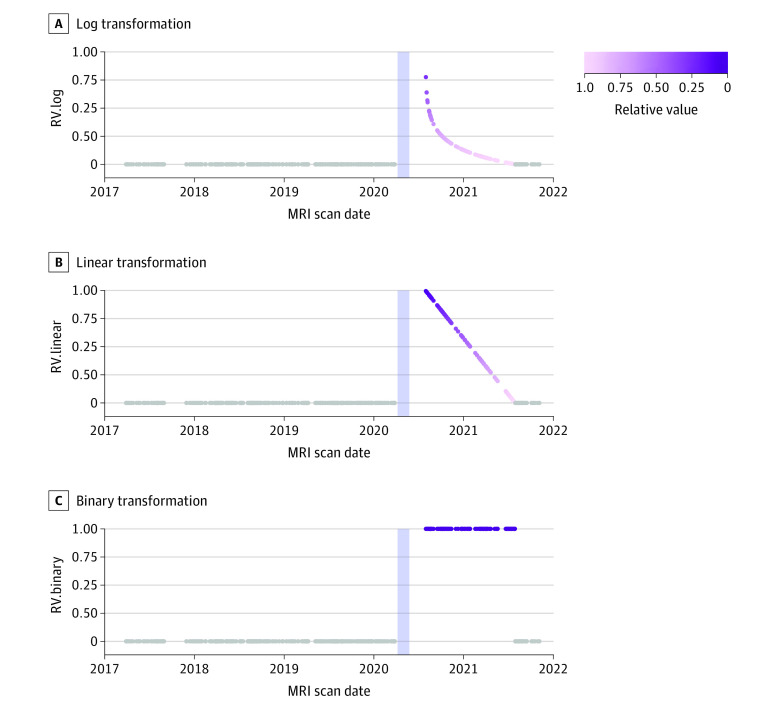
Transformations of Magnetic Resonance Imaging (MRI) Scan Date Relative to July 29, 2020, the dates of MRI scans taken from July 29, 2020, to July 29, 2021, were converted to relative values using the log (A), linear (B), and binary transformations (C) (ie, RV.log, RV.linear, and RV.binary), which are indicated by dots with color gradation from dark purple to light purple. In addition, based on our calculation method, MRI scans taken outside this year interval (both before and after) were set to 0, which are indicated by gray dots. The shaded area indicates the period of Japan's first state of emergency (April 7 to May 25, 2020).

To examine whether the hippocampus during the COVID-19 pandemic differed from that collected on other dates, we set a 1-year time interval (365 days) from July 29, 2020, to July 29, 2021 (including 141 magnetic resonance imaging [MRI] scans), after the first SoE (April 7 to May 25, 2020). Relative to July 29, 2020, the dates of MRI scans during this 1-year interval were converted into relative values (RVs) using the log, linear, and binary transformations. Specifically, we hypothesized that the extent of SoE-associated structural changes in the hippocampus would decrease with time following a logistic S-curve (Figure 2A) because the psychosocial effect of SoE on adolescents gradually died down. For more detailed information regarding the log transformation, see eMethods 8 in Supplement 1. To confirm this hypothesis, we additionally tested the linear hypothesis assuming the association of SoE with hippocampal structure would decrease with time linearly (Figure 2B) and the binary hypothesis assuming the scans collected during the 1-year interval were equally associated with the SoE (Figure 2C). Subsequently, the relative values using the log, linear, and binary transformations, denoted as RV.log, RV.linear, and RV.binary (as linear variables), were entered to the GAMMs or GLMMs to examine how the SoE was associated with the macrostructures and microstructures of the hippocampus. We included age as a smooth term using a penalized cubic regression spline and a basis function of 4, sex, SES, IQ, and ICV as linear terms, as well as a tensor interaction term between age and sex in the GAMMs. Additionally, the participant identifier was introduced as the random intercept. Therefore, for the mean hippocampal volume from 1060 scans as the primary outcome, we used 3 equations to find which hypothesis fitted most to the volumetric changes (eMethods 7 in Supplement 1). To confirm the GAMM, we also tested a GLMM for 141 scans that were scanned during the 1-year interval (July 29, 2020, to July 29, 2021) and 146 corresponding scans which were scanned at the age of 16 years or older, and their respective scans from the previous wave, including SoE and SoE × age interaction as variables, and the same covariates as the GAMM (eMethods 7 in Supplement 1). We conducted a power analysis for this GLMM using the simr package^24^ to check whether the SoE × age interaction had sufficient statistical power.

For hippocampal subfield volumes and DKI indices as the secondary outcomes, we used GLMMs to examine the associations between these outcomes and SoE (eMethods 7 in Supplement 1). For β coefficients of variables, 95% CIs were computed using a Wald *t* distribution approximation. To account for multiple comparisons across the 12 subfield volumes and 7 DKI indices, false-discovery rate (FDR) correction using Benjamini-Hochberg criterion (*q* = .05) was used. Moreover, a series of sensitivity analyses were conducted to investigate the outcomes of missing data on the primary results associated with mean hippocampal volume and the association between behavioral measurements and mean hippocampal volume (eMethods 9 in Supplement 1). Data were analyzed from August 2022 to December 2023.

## Results

### Demographic Characteristics

**Figure 3.**
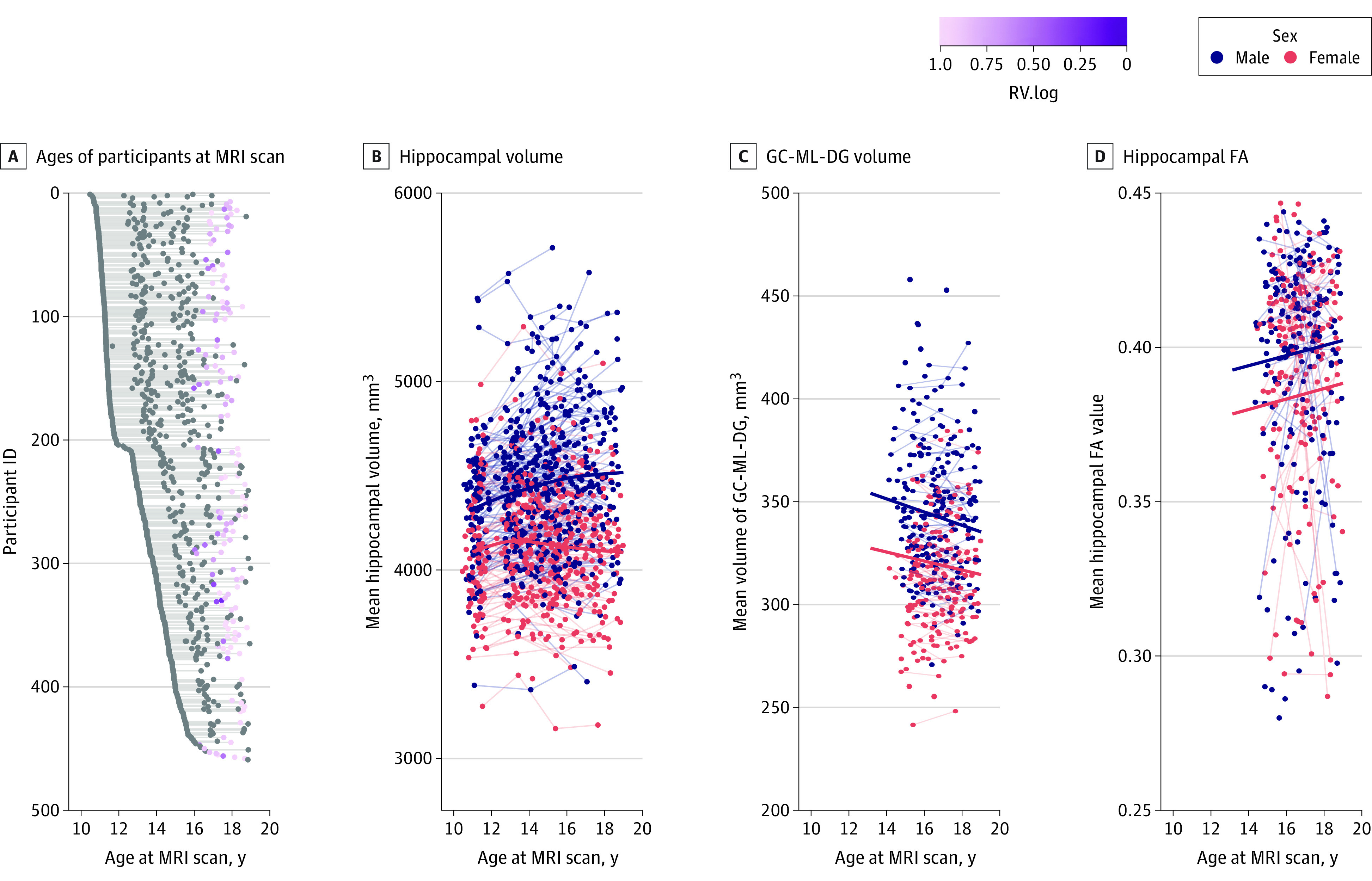
Longitudinal Sample and Developmental Trajectory of the Hippocampus During Adolescence Ages of participants at magnetic resonance imaging (MRI) scan (A), mean hippocampal volume (B), mean granule cell and molecular layer of the dentate gyrus (GC-ML-DG) volume (C), and mean hippocampal fractional anisotropy (FA) (D). ID indicates identifier.

From October 2013 to November 2021, we collected 1149 scans from 479 participants, of whom 336 had undergone 2 or more MRI scans, from 3 municipalities in the metropolitan area of Tokyo. Of these, 1060 scans from 459 participants (214 female participants [47%]), including 246 participants from wave 1 (median [IQR] age, 11.3 [11.1-11.7] years), 358 from wave 2 (median [IQR] age, 13.8 [13.3-14.5] years), 304 from wave 3 (median [IQR] age, 15.9 [15.4-16.5] years), and 152 from wave 4 (median [IQR] age, 17.9 [17.5-18.4] years) passed quality control procedures (eMethods 10 in Supplement 1) and were further analyzed (Figure 3A). The full demographic characteristics of the participants for each wave are presented in the Table.

**Table.** Participant Characteristics

Characteristic	pn-TTC participants at age 10			pn-TTC participants by wave, No. (%)			
	No pn-TTC measures (n = 2715)	pn-TTC measures (n = 456)	*P* value	Wave 1 (n = 246)	Wave 2 (n = 358)	Wave 3 (n = 304)	Wave 4 (n = 152)
Age, median (IQR), y	10.2 (10.0-10.3)	10.2 (10.0-10.3)	.45	11.3 (11.1-11.7)	13.8 (13.3-14.5)	15.9 (15.4-16.5)	17.9 (17.5-18.4)
Sex							
Male	1440 (53.0)	244 (53.5)	.89	132 (53.7)	193 (53.9)	165 (54.3)	75 (49.3)
Female	1275 (47.0)	212 (46.5)		114 (46.3)	165 (46.1)	139 (45.7)	77 (50.7)
Intelligence Quotient, median (IQR)	106.5 (98.0-117.1)	107.9 (101.2-117.4)	.02	NA	NA	NA	NA
Education level							
Elementary school, grade 5	NA	NA	NA	45 (18.3)	NA	NA	NA
Elementary school, grade 6	NA	NA	NA	159 (64.6)	1 (0.3)	NA	NA
Middle school, grade 1	NA	NA	NA	26 (10.6)	41 (11.5)	NA	NA
Middle school, grade 2	NA	NA	NA	16 (6.5)	160 (44.7)	NA	NA
Middle school, grade 3	NA	NA	NA	NA	120 (33.5)	41 (13.5)	NA
High school, grade 1	NA	NA	NA	NA	34 (9.5)	131 (43.1)	NA
High school, grade 2	NA	NA	NA	NA	2 (0.6)	102 (33.6)	14 (9.2)
High school, grade 3	NA	NA	NA	NA	NA	30 (9.9)	72 (47.4)
College, grade 1	NA	NA	NA	NA	NA	NA	66 (43.4)
Socioeconomic status							
Junior high school	12 (0.4)	0	.68	NA	NA	NA	NA
High school	212 (7.8)	39 (8.6)		NA	NA	NA	NA
2-y college	557 (20.5)	92 (20.2)		NA	NA	NA	NA
4-y university	1567 (57.7)	268 (58.8)		NA	NA	NA	NA
Graduate university	364 (13.4)	57 (12.5)		NA	NA	NA	NA
NA	3 (0.1)	0		NA	NA	NA	NA

Abbreviations: NA, not applicable; pn-TTC, population-neuroscience Tokyo TEEN Cohort.

### Developmental Trajectory of the Hippocampus During Adolescence

The GAMM showed an association with sex (β = −164.84; 95% CI, −221.58 to −108.09; *P* < .001; standardized β = −0.44; 95% CI, −0.59 to −0.29) and a nonlinear association with age for the mean hippocampal volume (effective degrees of freedom [EDF] = 2.81; reference degrees of freedom [RDF] = 3.40; *P* < .001). In addition, we found an age × sex significant interaction for boys (EDF = 1.43; RDF = 1.57; *P* = .002) but not for girls (EDF = 1.71; RDF = 2.12; *P* = .13) as shown in Figure 3B.

For hippocampal subfield volumes, GLMMs showed significant associations with age for 11 hippocampal subfields and with sex for 10 hippocampal subfields (Figure 3C and eFigure 1 in Supplement 1). The age × sex interactions were found in 3 subfields (CA1, CA2/3, and subiculum) but were not significant after FDR correction. All statistical results are summarized in eTable 1 in Supplement 1.

For all 7 microstructural indices, GLMMs showed associations with age for AK, MK, RK, and with sex for FA, AK, MK, and RK (all *P* < .05, FDR corrected; Figure 3D and eFigure 2 in Supplement 1). An age × sex interaction for AK was found only when FDR correction was not applied. All statistical results are summarized in eTable 2 in Supplement 1.

### Difference in Hippocampal Volume

**Figure 4.**
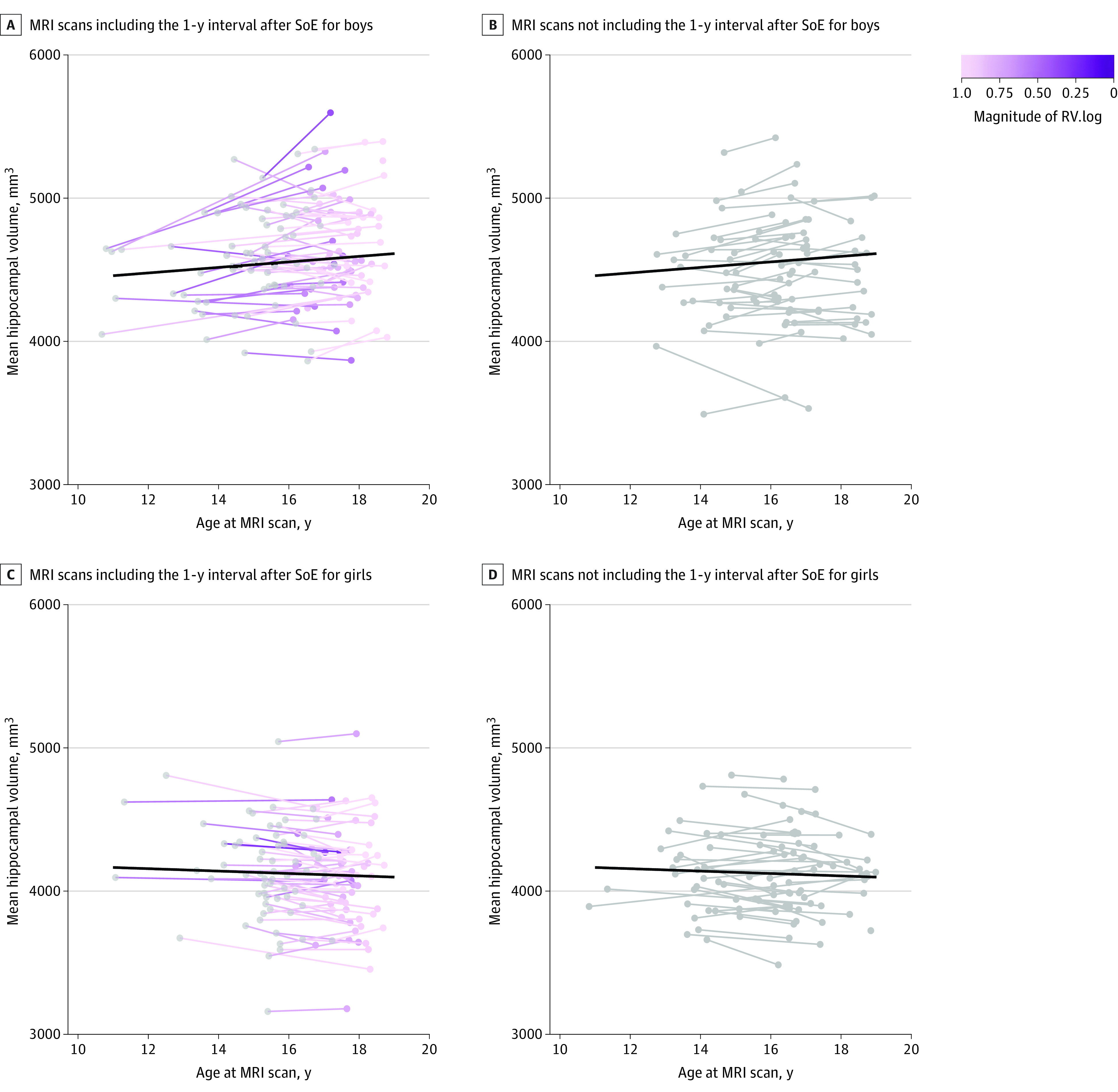
Association of the COVID-19 State of Emergency (SoE) With Mean Hippocampal Volume Along With Adolescent Developmental Trajectory Purple gradation indicates the magnitude of RV.log from 141 magnetic resonance imaging (MRI) scans for boys (A) and girls (C). The scans from the previous wave for the participants are shown in gray. The corresponding 146 MRI scans were scanned at the age of 16 years or older for boys (B) and girls (D).

When adding RV.log as a linear variable to the GAMM, we found that there was a significant association of SoE with the mean hippocampal volume (β = 102.19; 95% CI, 0.61 to 203.77; *P* = .049; standardized β = 0.02; 95% CI, 0.00 to 0.04). Using 141 scans that were scanned during the 1-year interval (July 29, 2020, to July 29, 2021) and 146 corresponding scans along with their respective previous scans, the confirmatory GLMM showed a significant SoE × age interaction (β = 129.62; 95% CI, 48.16 to 211.08; *P* = .002; standardized β = 0.04; 95% CI, 0.01 to 0.06) but not an association of SoE (β = −17.72; 95% CI, −263.84 to 228.40; *P* = .89; standardized β = 0.03; 95% CI, −0.06 to 0.13) with the mean hippocampal volume (Figure 4). Moreover, the likelihood ratio test based on 1000 simulations showed that the analysis achieved a power of 89.50% (95% CI, 87.43% to 91.33%) in detecting this 2-way interaction. Results in sensitivity analyses are provided in eAppendix 2 in Supplement 1. When examining the association of SoE with mean hippocampal volume using RV.linear and RV.binary, there were no significant results for either binary (β = 1.57; 95% CI, −28.90 to 32.05; *P* = .92; standardized β = 0.00; 95% CI, −0.03 to 0.03) or linear transformations (β = 19.23; 95% CI, −22.87 to 61.33; *P* = .37; standardized β = 0.01; 95% CI, −0.01 to 0.03).

### Differences in Subfield Volumes and Microstructure of Hippocampus

The GLMMs including RV.log as the variable showed associations of SoE with mean volumes in GC-ML-DG (β = 18.19; 95% CI, 2.97-33.41; uncorrected *P* = .02; standardized β = 0.06; 95% CI, 0.01-0.12); CA4: β = 12.75; 95% CI, 0.38-25.12; uncorrected *P* = .04; standardized β = 0.06; 95% CI, 0.00-0.11), and HATA (β = 5.67; 95% CI, 1.18-10.17; uncorrected *P* = .01; standardized β = 0.08; 95% CI, 0.02-0.15). There was no association of SoE with other subfield volumes (eTable 3 in Supplement 1).

An association of SoE with the FA values in the mean hippocampus was found (β = 0.03; 95% CI, 0.00-0.06; uncorrected *P* = .04; standardized β = 0.11; 95% CI, 0.01-0.21). Other indices were not significant (eTable 4 in Supplement 1).

## Discussion

The current study demonstrated that the first SoE declared for the COVID-19 pandemic was associated with a volumetric increase of the whole hippocampus during adolescent development. Increased subfield volumes in the GC-ML-DG, CA4, and HATA and increased hippocampal microstructural integration were also observed; however, these findings are less robust due to the potential influence of the multiple comparison problem. Nevertheless, all findings collectively demonstrate that a major life event may impact the development of hippocampal structure during middle adolescence, at least at the whole hippocampus level, further suggesting the plasticity and vulnerability of the adolescent hippocampus.

We clarified the developmental trajectory of the hippocampus to distinguish the typical hippocampus development from the changes associated with the COVID-19 pandemic. We found sex-specific nonlinear trajectories in hippocampal volume, and most hippocampal subfield volumes decreased from ages approximately 14 to 19 years, which are similar to those of previous studies.^25,26^ The associations between microstructural indices of the hippocampus and age were only found for MK, AK, and RK, which suggested that the 3 kurtosis parameters showed improved sensitivity and specificity in detecting developmental changes in neural tissues.^27,28^

To our knowledge, only 2 recent studies have provided direct evidence that adolescents had a larger hippocampal volume during the COVID-19 pandemic than before it.^11,12^ Although the authors claimed that the COVID-19 pandemic accelerated adolescent brain maturation, they could not show the direct relationship between increased hippocampal volume and psychological conditions. Given that the COVID-19 pandemic can be considered a potentially traumatic event for many individuals, posttraumatic stress disorder (PTSD) research could be used as indirect evidence to help us understand the action mechanism of the COVID-19 pandemic on the hippocampal structure. Many studies have shown that patients with PTSD who had been exposed to inherently stressful traumatic events always had a smaller hippocampal volume compared with healthy controls.^29,30^ Furthermore, higher levels of perceived stress were associated with a decreased adult or adolescent hippocampal volume.^31,32^ Hence, stressful experiences accompanying the COVID-19 pandemic may be associated with hippocampal volume atrophy. Counterintuitively, adolescents in our study seemed to have relatively low stress levels during the first SoE, consistent with similar findings in China where adolescents had fewer stressors during the school closure.^33,34^ Thus, we speculated that the increased hippocampal volume might be associated with stress reduction in adolescents. Discussion about hippocampal subfield volumes and microstructural integration is provided in eAppendix 3 in Supplement 1.

Since the alterations in the hippocampal structures were based on the log transformation for MRI collection dates after the SoE, the association of SoE with the hippocampal structures might be transient. Prior animal studies suggested the effects of stress on hippocampal dendritic structures are largely reversible, such as dendritic branching patterns taking 25 days to return to prestress levels after cessation of chronic stress for adolescents.^9,35^ Furthermore, the strength and duration of stress are highly associated with hippocampal structural changes.^36^ Since MRI acquisition restarted from July 31, 2020, we could not collect data from numerous participants in a short period of time. The continued low stress level during the SoE and few intermittent MRI scans after recovery of data collection, as well as the reversibility of the hippocampus, allowed us to hypothesize that the association of SoE with hippocampal structures should follow an exponential decay function. Additionally, as expected, binary and linear functions could not capture subtle changes in hippocampal structures. Therefore, our findings suggest that the reversibility of the hippocampal plasticity may allow us to continue to delineate the longitudinal normative development of the hippocampus.

### Limitations

This study had several limitations (for detailed limitations, see eAppendix 4 in Supplement 1). First, the finding that there are no significant associations between psychological distress and hippocampal volume prevents us from asserting that structural changes in the hippocampus are only associated with stress. Second, changes in the environmental complexity during the SoE relative to those in other periods were not monitored in this study. More well-planned behavioral measurements in future studies may help draw confirmative conclusions about the mechanism of action of a major global life event. Third, our main significant findings were based on the complete-case analysis strategy. The lack of significant results derived from multiple imputation may not be considered entirely convincing because this method may not be appropriate for situations where data missingness exceeds 50%.^37^ Fourth, for hippocampal subfield volumes and hippocampal microstructures, a smaller sample size and a narrower age range may result in nonsignificance after FDR correction. Fifth, this study used automated hippocampal subfield segmentation using FreeSurfer version 6.0.0 instead of another popular tool, volBrain, known for its shorter processing time. Moreover, a comparative study demonstrated an association in terms of hippocampal subfield volumes between these 2 segmentation tools.^38^ Recently, more advanced methodologies, such as machine learning, may offer improved segmentation accuracy.^39^ Additionally, the adolescents in the pn-TTC are Tokyo residents. Caution is needed in directly generalizing these findings to adolescents in rural areas or from low SES families in urban settings.

## Conclusions

Our findings revealed that Japan’s first SoE declared for the COVID-19 pandemic was associated with a transient increase in the whole hippocampal volume in adolescents. Increases in hippocampal subfields and microstructural integration associated with the SoE are not conclusive due to a lack of samples. In summary, this study suggests that a major life event might alter the developmental trajectory of the adolescent hippocampus. Moreover, since adolescents in the pn-TTC are from the metropolitan area of Tokyo and were influenced by local policies addressing the COVID-19 pandemic, future research should examine whether similar transient hippocampal structural changes occur in other populations across different countries.

## Notes

Supplement 1. **eMethods 1. **Behavioral Measurements and Statistical Analysis **eAppendix 1.** Supplementary Results 1: Disruptions in Daily Routines During the SoE Period **eMethods 2. **Eligibility Criteria **eMethods 3. **MRI Acquisition Parameters **eMethods 4. **Traveling Subject Harmonization **eMethods 5. **Image Preprocessing and Hippocampal Segmentation **eMethods 6. **Diffusion Kurtosis Imaging **eMethods 7. **Statistical Equations **eMethods 8. **Statistical Analysis **eMethods 9. **Sensitivity Analyses **eMethods 10. **Quality Control **eFigure 1.** Developmental Trajectory of 12 Hippocampal Subfield Volumes During Adolescence **eTable 1. **Developmental Trajectory of Hippocampal Subfield Volume **eFigure 2. **Developmental Trajectory of the Hippocampal Microstructure During Adolescence **eTable 2. **Developmental Trajectory of Hippocampal Microstructure **eAppendix 2.** Supplementary Results 2: Sensitivity Analyses **eTable 3. **The Effect of SoE on Hippocampal Subfield Volumes **eTable 4. **The Effect of SoE on the Microstructure of the Hippocampus **eAppendix 3.** Supplementary Discussion **eAppendix 4. **Supplementary Limitations
eReferences

Supplement 2.
Data Sharing Statement
