# Supplementary material for: Hippocampal Structures Among Japanese Adolescents Before and After the COVID-19 Pandemic
Source: JAMA Netw Open. 2024 Feb 8;7(2):e2355292. doi: 10.1001/jamanetworkopen.2023.55292 (PMC10853829; doi:10.1001/jamanetworkopen.2023.55292)
Supplement: Supplement 1. — eMethods 1. Behavioral Measurements and Statistical Analysis eAppendix 1. Supplementary Results 1: Disruptions in Daily Routines During the SoE Period eMethods 2. Eligibility Criteria eMethods 3. MRI Acquisition Parameters eMethods 4. Traveling Subject Harmonization eMethods 5. Image Preprocessing and Hippocampal Segmentation eMethods 6. Diffusion Kurtosis Imaging eMethods 7. Statistical Equations eMethods 8. Statistical Analysis eMethods 9. Sensitivity Analyses eMethods 10. Quality Control eFigure 1. Developmental Trajectory of 12 Hippocampal Subfield Volumes During Adolescence eTable 1. Developmental Trajectory of Hippocampal Subfield Volume eFigure 2. Developmental Trajectory of the Hippocampal Microstructure During Adolescence eTable 2. Developmental Trajectory of Hippocampal Microstructure eAppendix 2. Supplementary Results 2: Sensitivity Analyses eTable 3. The Effect of SoE on Hippocampal Subfield Volumes eTable 4. The Effect of SoE on the Microstructure of the Hippocampus eAppendix 3. Supplementary Discussion eAppendix 4. Supplementary Limitations eReferences [file jamanetwopen-e2355292-s001.pdf]

Supplemental Online Content

Cai L, Maikusa N, Zhu Y. Hippocampal structures among Japanese adolescents before and after the COVID-19 pandemic. *JAMA Netw Open*. 2024;7(2):e2355292. doi:10.1001/jamanetworkopen.2023.55292

eMethods 1. Behavioral Measurements and Statistical Analysis

eAppendix 1. Supplementary Results 1: Disruptions in Daily Routines During the SoE Period

eMethods 2. Eligibility Criteria

eMethods 3. MRI Acquisition Parameters

eMethods 4. Traveling Subject Harmonization

eMethods 5. Image Preprocessing and Hippocampal Segmentation

eMethods 6. Diffusion Kurtosis Imaging

eMethods 7. Statistical Equations

eMethods 8. Statistical Analysis

eMethods 9. Sensitivity Analyses

eMethods 10. Quality Control

eFigure 1. Developmental Trajectory of 12 Hippocampal Subfield Volumes During Adolescence

eTable 1. Developmental Trajectory of Hippocampal Subfield Volume

eFigure 2. Developmental Trajectory of the Hippocampal Microstructure During Adolescence

eTable 2. Developmental Trajectory of Hippocampal Microstructure

eAppendix 2. Supplementary Results 2: Sensitivity Analyses

eTable 3. The Effect of SoE on Hippocampal Subfield Volumes

eTable 4. The Effect of SoE on the Microstructure of the Hippocampus

eAppendix 3. Supplementary Discussion

eAppendix 4. Supplementary Limitations

eReferences

This supplemental material has been provided by the authors to give readers additional information about their work.

© 2024 Cai et al. *JAMA Network Open.*

eMethods 1. Behavioral Measurements and Statistical Analysis

Weekly time spent at home in Tokyo, Japan

Google provides the COVID-19 Community Mobility Reports for different countries and regions, which use anonymous, aggregated geolocation data from mobile phones to chart movement trends over time (https://www.google.com/covid19/mobility/). We selected the specific region (Tokyo, Japan), time range (2020/02/15-2020/10/31), and variable (Stay.Home.ave.week) from the COVID-19 Community Mobility Reports. The variable Stay.Home.ave.week describes the week mean of the daily time spent at home (unit: hours).

Web-based questionnaires in general adolescents

Of the 479 population-neuroscience Tokyo TEEN Cohort (pn-TTC) participants, 90 agreed to participate in a web-based questionnaire every month. The participation periods varied from July 2019 to October 2022. The web-based questionnaire included measurements of *weekly activities* and *psychological distress*. Between November 2019 and October 2020, 516 responses from 65 participants were analyzed (mean ± SD = 7.9 ± 3.5; range: 1-12).

Weekly activities

The history of weekly activities in the past 30 days was asked for (1) the weekly number of days at school and (2) weekly extracurricular activity days. The response options are as follows: (a) In the last month, on average, how many days per week did you go to school? (b) In the last month, on average, how many days per week did you participate in regular extracurricular activities such as tutoring school, club activities, and part-time jobs?

Psychological distress

Psychological distress was assessed using the Japanese version of the K6, a scale that examines psychological distress over the past 30 day.[1] The validity of the Japanese version was confirmed.[2] The K6 is a six-item self-report measure asking participants how often they felt nervous, hopeless, restless, so depressed that nothing could cheer them up, that everything was an effort, and worthless in the past 30 days. Each response is scored from 0 (never) to 4 (always), and the total score for 6 items (0 to 24) is considered. The higher the total K6 score, the higher the psychological distress.

To examine whether Japan's first state of emergency (SoE) affected the mobility of citizens in Tokyo, we ran a general linear model (GLM) to compare the week mean of the daily time spent at home (that is, Stay.Home.ave.week) from each month during March 2020 - October 2020 with that in February 2019 as a reference month, respectively. Furthermore, to investigate whether the SoE affected the weekly number of days at school, weekly extracurricular activity days, and psychological distress for adolescents from

pn-TTC subsamples, we adopted the generalized linear mixed models (GLMMs) to compare the corresponding values from each month between December 2019 and October 2020 with those in November 2019 as a reference month, respectively. Participants entered the GLMMs as a random intercept effect.

© 2024 Cai et al. *JAMA Network Open.*

eAppendix 1. Supplementary Results 1: Disruptions in Daily Routines During the SoE Period

We found that, relative to February 2020, the week mean of the daily time spent at home by Tokyo citizens significantly increased for every month from March 2020 to October 2020 using the COVID-19 Community Mobility Reports (Fig 1B, β_{March} = 3.95, 95% CI [2.55, 5.35], $p < 0.001$, Std. beta = 2.78, 95% CI [2.54, 3.02]; β_{April} = 16.36, 95% CI [14.95, 17.77], $p < 0.001$, Std. beta = 3.40, 95% CI [3.16, 3.64]; β_{May} = 19.99, 95% CI [18.59, 21.39], $p < 0.001$, Std. beta = 1.79, 95% CI [1.55, 2.03]; β_{June} = 10.51, 95% CI [9.10, 11.92], $p < 0.001$, Std. beta = 0.67, 95% CI [0.43, 0.91]; β_{July} = 9.40, 95% CI [8.00, 10.80], $p < 0.001$, Std. beta = 1.65, 95% CI [1.41, 1.89]; β_{August} = 9.69, 95% CI [8.28, 11.09], $p < 0.001$, Std. beta = 1.33, 95% CI [1.09, 1.57]; $\beta_{September}$ = 7.83, 95% CI [6.42, 9.24], $p < 0.001$, Std. beta = 0.97, 95% CI [0.74, 1.21]; $\beta_{October}$ = 5.72, 95% CI [4.32, 7.12], $p < 0.001$, Std. beta = 1.60, 95% CI [1.36, 1.84]).

When compared with the weekly number of days at school from the reference month (November 2019), 65 adolescents from the pnTTC subsample showed a significantly reduced weekly number of days at school for January, March, April, May, June, and August 2020 (Fig 1D, $\beta_{January}$ = -0.77, 95% CI [-1.40, -0.13], p = 0.02, Std. beta = -0.12, 95% CI [-0.38, 0.14]; β_{March} = -1.83, 95% CI [-2.47, -1.20], $p < 0.001$, Std. beta = -0.02, 95% CI [-0.30, 0.25]; β_{April} = -5.37, 95% CI [-6.00, -4.75], $p < 0.001$, Std. beta = -2.25, 95% CI [-2.51, -1.99]; β_{May} = -5.53, 95% CI [-6.17, -4.90], $p < 0.001$, Std. beta = -1.26, 95% CI [-1.51, -1.01]; β_{June} = -3.10, 95% CI [-3.71, -2.50], $p < 0.001$, Std. beta = -0.24, 95% CI [-0.49, 0.02]; β_{August} = -2.84, 95% CI [-3.47, -2.22], $p < 0.001$, Std. beta = -1.16, 95% CI [-1.41, -0.90]). Similarly, the reduced weekly extracurricular activity days were found for March, April, May, June, and July 2020 (Fig 1E, β_{March} = -0.87, 95% CI [-1.63, -0.11], p = 0.03, Std. beta = -0.04, 95% CI [-0.40, 0.33]; β_{April} = -2.40, 95% CI [-3.15, -1.66], $p < 0.001$, Std. beta = -1.17, 95% CI [-1.51, -0.82]; β_{May} = -2.54, 95% CI [-3.30, -1.78], $p < 0.001$, Std. beta = -0.89, 95% CI [-1.22, -0.55]; β_{June} = -1.93, 95% CI [-2.66, -1.20], $p < 0.001$, Std. beta = -0.52, 95% CI [-0.87, -0.18]; β_{July} = -1.14, 95% CI [-1.89, -0.39], p = 0.003, Std. beta = -1.10, 95% CI [-1.45, -0.76]). Moreover, the psychological distress of the adolescents during April, June, and July (Fig. 1F, β_{April} = -1.51, 95% CI [-2.57, -0.46], p = 0.005, Std. beta = -0.23, 95% CI [-0.50, 0.05]; β_{June} = -1.06, 95% CI [-2.09, -0.03], p = 0.04, Std. beta = -0.31, 95% CI [-0.58, -0.04]; β_{July} = -1.20, 95% CI [-2.25, -0.14], p = 0.03, Std. beta = -0.39, 95% CI [-0.66, -0.12]) was lower than that in the reference month (November 2019).

© 2024 Cai et al. *JAMA Network Open.*

eMethods 2. Eligibility Criteria

Prior to participation in the pn-TTC wave 1, the following exclusion criteria were applied: (i) current problems in mental health, interpersonal relationships, or behaviors; (ii) visual or hearing disabilities; (iii) history of head injury accompanied by more than 5 min of loss of consciousness; (iv) current chronic endocrine disease or metabolic disease; and (v) current use of medication that affect the central nervous system. The participants in the follow-up waves 2, 3, and 4 of the pn-TTC study were primarily drawn from individuals who had participated in the previous wave, showed no abnormal brain MRI results, and met the exclusion criteria. To achieve the anticipated recruitment number (approximately 300), additional participants were recruited from the TTC study, applying the same exclusion criteria as in pn-TTC wave 1. For participants scanned after July 2020, all participants self-reported on the day of the MRI scan that they did not exhibit any symptoms of COVID-19 and had not been diagnosed as carriers of the virus. Additionally, before MRI scanning, we measured their body temperature to ensure that all participants fell within the normal range. As estimated, there were 2,197,533 10-20-year-olds in Tokyo in 2020. The infection rate was 0.00291236% during the first SoE, while 0.00428079% during 1 year after the SoE. Thus, adolescents in the pn-TTC should have extremely low probability of being diagnosed as carriers of the virus.

© 2024 Cai et al. *JAMA Network Open.*

eMethods 3. MRI Acquisition Parameters

For the four waves of data collection, two 3-T scanners and three acquisition procedures were performed. Procedure 1 (corresponding to wave 1 and the main part of wave 2) used a Philips Achieva scanner (Philips Medical Systems, Best, The Netherlands) with an 8-channel head coil and obtained 522 scans. Procedure 2 (corresponding to a small remaining part of wave 2) used a Siemens Prisma scanner (Siemens Healthineers, Erlangen, Germany) with a 64-channel head coil and obtained 121 scans. Procedure 3 used a Siemens Prisma scanner with a 32-channel head coil and obtained 506 scans. For Procedure 1, sagittal T1-weighted images were acquired using a 3D magnetization-prepared rapid gradient echo (MPRAGE) sequence with the following parameters: repetition time (TR) = 7.0 ms; echo time (TE) = 3.2 ms; flip angle = 9°; field of view (FOV) = 256 mm × 240 mm × 200 mm; voxel size = 1 × 1 × 1.2 mm. For Procedure 2, sagittal T1-weighted images were acquired using the 3D MPRAGE sequence with the following parameters: TR = 1900 ms; TE = 2.53 ms; flip angle = 9°; FOV = 256 mm × 256 mm × 256 mm; voxel size = 1 × 1 × 1.2mm. The protocol for Procedure 3 was based on the Human Connectome Project (HCP) for lifespan development and aging projects.[3,4] This protocol consisted of T1-weighted, T2-weighted, and multi-shell diffusion MRI. T1-weighted images were acquired using the MPRAGE sequence with the following parameters: TR = 2400 ms; TE = 2.22 ms; flip angle = 8°; FOV = 208 mm × 300 mm × 320 mm; voxel size = 0.8 mm isotropic. The T2-weighted images were acquired using an SPC sequence (TR = 3200 ms, TE = 563 ms, flip angle = 120°, FOV = 208 mm × 300 mm × 320 mm; voxel size = 0.8 mm isotropic). For diffusion MRI acquisition in Procedure 3, diffusion weightings of b = 200, 500, 1500, and 3000 s/mm^2 were applied in 3, 6, 46, and 46 directions, respectively. Seven images without diffusion-sensitizing gradients (i.e., “b0 images”) were acquired. The remaining acquisition parameters were as follows: voxel size = 1.5 mm isotropic; multi-band acceleration factor = 4; TR = 3230 ms; TE= 89.2 ms; flip angle = 78°; FOV = 140 mm × 140 mm × 92 mm; bandwidth = 1700 Hz). In addition, a pair of reverse phase-encoded spin-echo field maps (anterior to posterior, and posterior to anterior) was also collected to aid distortion correction in preprocessing (voxel size = 2 mm isotropic, TR = 8000 ms, TE = 66 ms, flip angle = 90°, FOV = 104 mm × 104 mm × 72 mm, bandwidth = 2290 Hz).

© 2024 Cai et al. *JAMA Network Open.*

eMethods 4. Traveling Subject (TS) Harmonization

To remove the difference in data collection sites among the four waves of pn-TTC, we followed the TS harmonization method,[5,6] which extends the GLM harmonization model using a TS dataset. The TS harmonization model can be described as follows:

$$y(i,j,v) \;=\; X_s^T(i,j)\beta_s(v) \;+\; X_p^T(i,j)\beta_p(v) \;+\varepsilon(i,j,v),$$

where $\beta_p(v)$ represents the participant factor, and X_p (i, j) is the n × 1 vector of the participant indicator. $\beta_s(v)$ represents the coefficient of the site factor, namely the measurement bias, and X_s (i, j) is the k × 1 vector of the site indicator. To estimate the respective parameters, we calculated the inverse matrix for X_p (i, j) and X_s (i, j). In this study, all participants were healthy and identical at each site; therefore, sampling bias was not considered. However, the design matrix of the GLM was rank-deficient; thus, we used the Moore–Penrose pseudo-inverse matrix via the “pinv” function in MATLAB (R2020b) to estimate $\widehat{\beta_s}(v)$ and $\widehat{\beta_p}(v)$. After estimating, $\widehat{\beta_s}(v)$, the harmonized hippocampal volumes were set as follows:

$$y^{TS\,glm}(i,j,v) \;= y(i,j,v) \;- X_s^T(i,j)\hat{\beta}_s(v)\;.$$

© 2024 Cai et al. *JAMA Network Open.*

eMethods 5. Image Preprocessing and Hippocampal Segmentation

The T1-weighted images obtained using Procedures 1 and 2 were preprocessed using the legacy style in the HCP pipeline version 4.3,[8] primarily via the FreeSurfer v6.0.0 "recon-all" command. The images obtained using Procedure 3 were preprocessed using the standard style of the HCP pipeline,[7] which requires both T1-weighted and T2-weighted images. However, if T2-weighted images were unavailable for any reason, we preprocessed only T1-weighted images using FreeSurfer "recon-all" similar to preprocessing steps for Procedures 1 and 2. Intracranial volumes were obtained using segmentation-based intracranial volume (ICV) in FreeSurfer. Finally, bilateral hippocampal volumes were extracted from the "Left-Hippocampus" and "Right-Hippocampus" fields in FreeSurfer's aseg.stats file. Extracted hippocampal volumes were harmonized using the TS harmonization method to remove differences in the data collection sites among the four waves (see eMethods 4: Traveling subject (TS) harmonization).[5,6] Similar to previous studies,[8,9] we computed the averaged hippocampal volumes across two hemispheres as an index in the statistical analysis of the Main text. In addition, hippocampal subfield volumes were obtained using the hippocampal subfield segmentation algorithm in FreeSurfer v6.0.0 based on a statistical atlas built primarily upon ultra-high resolution (approximately 0.1 mm isotropic) ex vivo MRI data.[10] Considering that the use of T1-weighted images at standard resolution relies heavily on prior information of the atlas and hardly reflects the underlying neurobiological complex structure of the hippocampus, both T1-weighted and T2-weighted images were used as input to obtain volumes for 12 subfield regions, e.g., CA1, CA2/3, CA4, subiculum, the granule cell and molecular layer of the dentate gyrus (GC-ML-DG), hippocampus-amygdala transition area (HATA), hippocampal tail, hippocampal fissure, molecular layer, presubiculum, parasubiculum, and fimbria. For each scan, volumetric estimates for each subfield were extracted and averaged across hemispheres.

© 2024 Cai et al. *JAMA Network Open.*

eMethods 6. Diffusion Kurtosis Imaging (DKI)

The raw diffusion scans were preprocessed using the HCP diffusion preprocessing pipeline.[7] In brief, corrections for gradient distortion, static-field (B0) distortion and eddy current distortion, and cross modal registration were performed.[7,11] The intensity was normalized by the mean of volumes with b = 0 s/mm^2 (b0 volumes) and the B0-inhomogeneity distortion was corrected using two opposing phase encoded images and FSL's Topup.[12] The eddy current induced field inhomogeneities and the head motion for each image volume were corrected using FSL's Eddy,[13] followed by correction for the gradient nonlinearity. Diffusion data were registered to the structural T1-weighted AC-PC space using the b0 volume and the white surface using the BBR cost function in FSL and FreeSurfer's BBRegister. The diffusion gradient vectors were rotated based on the rotational information of the b0 to T1-weighted transformation matrix.

Subsequently, microstructural metrics were estimated using the DKI model,[14] as implemented in DIPY v1.5.0.[15] The DKI model could produce 4 conventional diffusion tensor imaging (DTI) images: fractional anisotropy (FA), mean diffusivity (MD), axial diffusivity (AD), and radial diffusivity (RD). The FA reflects the degree of diffusion anisotropy, and MD represents the average diffusivity in all directions. The AD reflects the diffusion parallel to the primary diffusion axis, and RD captures the diffusion perpendicular to the primary diffusion axis. In addition, the DKI model could provide 3 kurtosis images: mean kurtosis (MK), axial kurtosis (AK), radial kurtosis (RK).

To obtain the mean value of each microstructural metric in bilateral hippocampi, we first transformed whole brain segmentation images in native space to those in diffusion space using FSL Linear Image Registration Tool (FLIRT, https://fsl.fmrib.ox.ac.uk/fsl/fslwiki/FLIRT), and then extracted the hippocampal masks using FreeSurfer command ‘mri_binarize’ (https://surfer.nmr.mgh.harvard.edu/fswiki/mri_binarize). Finally, for each microstructural metric, the mean values of bilateral hippocampi were obtained using the FSL ‘fslstats’ tool, and further were averaged across hemispheres as an index in the statistical analysis of the Main text.

© 2024 Cai et al. *JAMA Network Open.*

eMethods 7. Statistical Equations

(1) GAMM: mean.hippo ~ s(c.age) + sex + ti(c.age, by=sex, bs='fs') + c.ICV + s(PID, bs = "re")

(2) GLMM: mean.var ~ c.age + sex + c.age:sex + c. ICV + (1 | PID)

(3) GAMM: mean.hippo ~ RV.log + s(c.age) + sex + ti(c.age, by=sex, bs='fs') + c.SES + c.IQ + c.ICV + s(PID, bs = "re")

(4) GAMM: mean.hippo ~ RV.linear + s(c.age) + sex + ti(c.age, by=sex, bs='fs') + c.SES + c.IQ + c.ICV + s(PID, bs = "re")

(5) GAMM: mean.hippo ~ RV.binary + s(c.age) + sex + ti(c.age, by=sex, bs='fs') + c.SES + c.IQ + c.ICV + s(PID, bs = "re")

(6) GLMM: mean.hippo ~ RV.log*c.age + c.age*sex + c.IQ + c.SES + c. ICV + (1 | PID)

(7) GLMM: mean.var ~ RV.log + c.age + sex + c.age:sex + c.IQ + c.SES + c. ICV + (1 | PID)

© 2024 Cai et al. *JAMA Network Open.*

eMethods 8. Statistical Analysis

To examine whether the hippocampus during the COVID-19 pandemic differed from that collected in other dates, we set a 1-year time interval (365 days) from July 29, 2020 to July 29, 2021 (including 141 MRI scans) after the first SoE (2020/04/07–2020/05/25). Relative to July 29, 2020, the dates of MRI scans during this 1-year interval were converted into relative values using the log, linear, and binary transformations. The selection of the starting and ending dates for this 1-year interval was based on the restart date of MRI scans (2020/07/31). Moreover, we speculated that SoE-related structural changes in the hippocampus might exhibit a time lag effect and an approximate 4-month interval (from 2020/04/07 to 2020/07/31) may allow us to observe structural changes in the hippocampus. Specifically, we hypothesized that the extent of SoE-related structural changes in the hippocampus declined with time following a logistic S-curve (Fig. 2A) because the psychosocial effect of SoE on adolescents gradually died down. As shown in Fig. 2A, if the MRI scan date is 2020/08/13, the relative value calculated by the log transformation is 0.48. However, if the MRI scan date is 2020/12/23, the relative value is 0.14. The magnitude of relative values is indicated by purple gradation, and MRI scans taken outside this 1-year interval (both before and after) were set to 0. In other words, the MRI scan date is closer to the predefined date (i.e., 2020/07/29), and the relative value calculated by the log transformation is larger. To confirm this hypothesis, we additionally tested the linear hypothesis assuming the effect of SoE on hippocampal structure declined with time linearly (Fig. 2B) and the binary hypothesis assuming the scans collected during the 1-year interval mentioned above were equally affected by the SoE (Fig. 2C). Subsequently, the relative values using the log, linear, and binary transformations, denoted as RV.log, RV.linear, and RV.binary (as linear predictors), entered to the GAMMs or GLMMs to examine how the SoE impacted the macro- and micro-structures of the hippocampus. We included age as a smooth term using a penalized cubic regression spline and a basis function of 4, sex, SES, IQ, and ICV as linear terms, as well as a tensor interaction term between age and sex in the GAMMs. Additionally, the participant ID (PID) was introduced as the random intercept. All numeric covariables (i.e., age, SES, IQ, ICV) were z-transformed (i.e., c.age, c.SES, c.IQ, c.ICV). Sex and the PID were treated as factors. Therefore, for the mean hippocampal volume from 1060 scans as the primary outcome, we used 3 equations to find which hypothesis fitted most to the volumetric changes (eMethods 7, equations 3-5, in Supplement). To confirm the GAMM, we also tested a GLMM for 141 scans that were scanned during the 1-year interval (2020/07/29–2021/07/29) and 146 corresponding scans which were scanned at the age of 16 years or older, and their respective scans from the previous one wave, including a main effect of SoE and SoE × age interaction as predictor variables, and the same covariates as the GAMM (eMethods 7, equation 6, in Supplement). We conducted a power analysis for this GLMM using the “simr” package to check whether the SoE × age interaction had sufficient statistical power.

© 2024 Cai et al. *JAMA Network Open.*

eMethods 9. Sensitivity Analyses

A series of sensitivity analyses were conducted. To address the potential bias due to missing values, we reapplied GAMM analysis to investigate the SoE effect on mean hippocampal volume using both a full four-wave dataset with whole-wave missingness and an imputed dataset. For the imputed dataset, we used the “mice” package to conduct multiple imputation for a full four-wave dataset with whole-wave missingness. Specifically, out of 1836 observations across all waves, 795 data points were missing for age, TIV, and mean hippocampal volume, and 462 were missing for RV.log. The predictive mean matching was used for these continuous variables. A total of 30 imputations were run, and the mean values across these imputations were used for the variables with missing data points.

Moreover, even though the very limited sample size, we attempted to analyze the relationship between behavioral measurements (i.e., psychological distress, weekly number of days at school, and weekly extracurricular activity days) and mean hippocampal volume. To explore the relationship between psychological distress, as assessed by K6 scores, and mean hippocampal volume, we initially calculated the average K6 scores for the 3 months preceding participants' MRI scanning. Among the 90 adolescents with K6 scores in the pn-TTC, only 27 participants (median [IQR] age, 17.6 [1.1] years; 16 [59.3%] girls) scanned from October 11, 2019, to August 27, 2021, contributed to further statistical analysis. Subsequently, we employed a GLM with mean hippocampal volume as the outcome variable, 3-month mean K6 scores as the predictor, and age, sex, age by sex interaction, SES, IQ, and TIV as covariates. Similarly, we also explored the relationships between the weekly number of days at school, weekly extracurricular activity days and mean hippocampal volume. Data from 27 participants (median [IQR] age, 17.7 [0.8] years; 16 [59.3%] girls) scanned from September 29, 2020, to October 12, 2021, was fed to two GLMs with the weekly number of days at school or weekly extracurricular activity days as the predictor. The covariables were age, sex, age by sex interaction, SES, IQ, and TIV.

© 2024 Cai et al. *JAMA Network Open.*

eMethods 10. Quality Control (QC)

Of 1,149 structural scans from 479 participants, 19 scans were excluded due to mass anatomical incidental findings or brain operation history, 40 scans were excluded based on a manual check for poor image quality by three trained staff, 19 scans were excluded because of a failure of image preprocessing, and 10 scans because of substantial outliers detected by the ENIGMA QC script. After QC steps, 1,060 scans from 459 participants were analyzed for further investigation. In 395 diffusion scans from 297 participants collected during waves 3 and 4, we used a quality control tool eddy_squad from FSL[16] and a visual check, to identify the data with poor quality. We excluded low-quality diffusion data with total outliers above 1%[17] or with excessive motion (average absolute motion > 10 mm)[18]. After diffusion QC, 382 diffusion scans from 288 participants were further analyzed.

© 2024 Cai et al. *JAMA Network Open.*

eFigure 1. Developmental Trajectory of 12 Hippocampal Subfield Volumes During Adolescence. Abbreviations: cornu ammonis (CA), the granule cell and molecular layer of the dentate gyrus (GC-ML-DG), hippocampus-amygdala transition area (HATA).

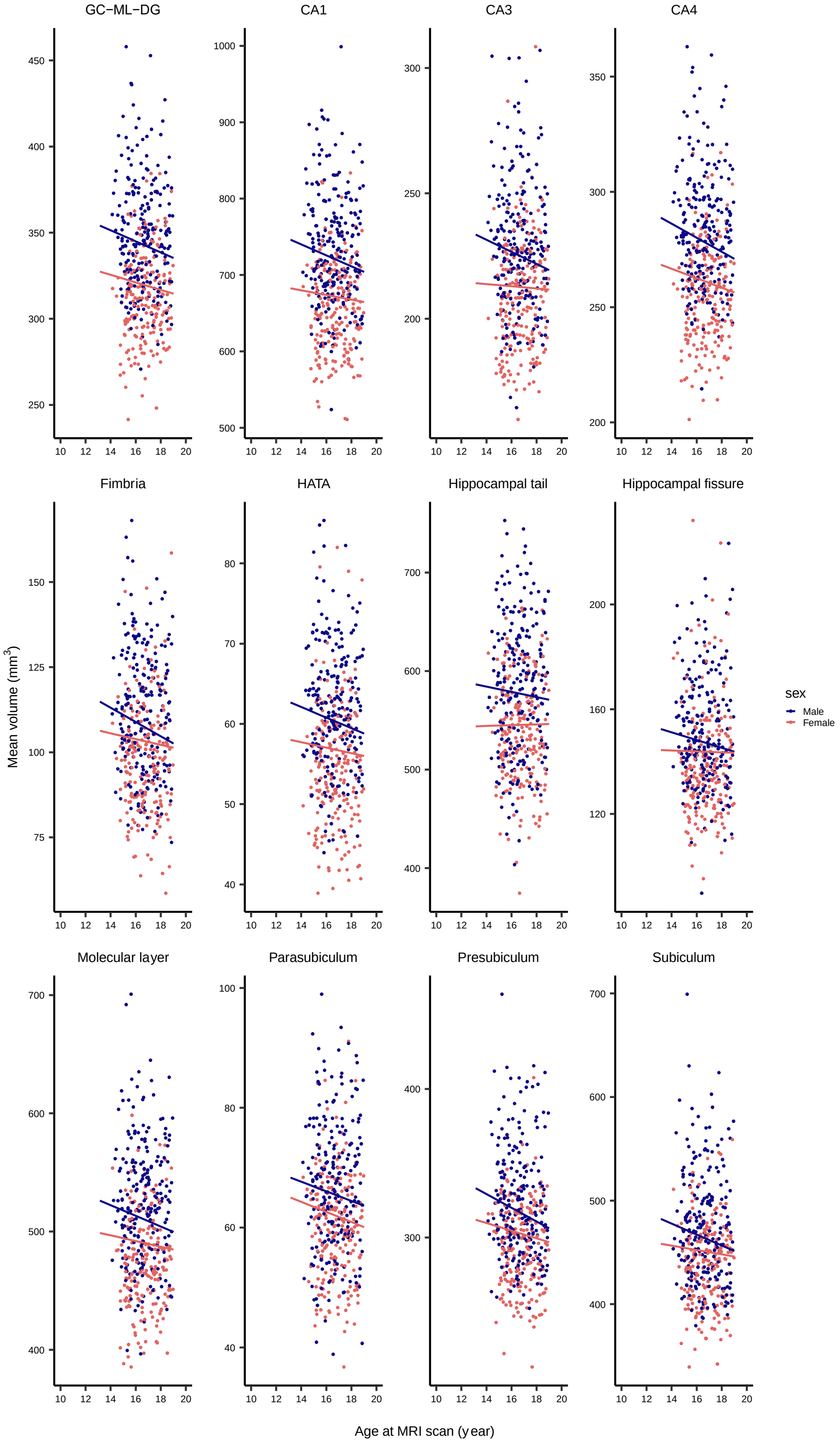

© 2024 Cai et al. *JAMA Network Open.*

eTable 1. Developmental Trajectory of Hippocampal Subfield Volume

Age						
Subfield	beta	95% CI	p	Std.beta	95% CI	FDR-p
Hippocampal_tail	-3.10	[-6.52, 0.32]	0.076	-0.05	[-0.10, 0.00]	0.076
**subiculum**	**-6.19**	**[-8.60, -3.77]**	**0.000**	**-0.12**	**[-0.17, -0.07]**	**0.000**
**CA1**	**-8.43**	**[-12.22, -4.65]**	**0.000**	**-0.11**	**[-0.16, -0.06]**	**0.000**
**hippocampal fissure**	**-1.73**	**[-3.11, -0.35]**	**0.014**	**-0.08**	**[-0.14, -0.02]**	**0.016**
**presubiculum**	**-5.41**	**[-7.38, -3.44]**	**0.000**	**-0.15**	**[-0.20, -0.09]**	**0.000**
**parasubiculum**	**-0.94**	**[-1.53, -0.35]**	**0.002**	**-0.09**	**[-0.14, -0.03]**	**0.003**
**molecular layer**	**-5.29**	**[-8.94, -1.63]**	**0.005**	**-0.10**	**[-0.17, -0.03]**	**0.006**
**GC-ML-DG**	**-3.76**	**[-5.42, -2.10]**	**0.000**	**-0.11**	**[-0.16, -0.06]**	**0.000**
**CA2/3**	**-2.83**	**[-4.34, -1.31]**	**0.000**	**-0.11**	**[-0.17, -0.05]**	**0.001**
**CA4**	**-3.58**	**[-4.96, -2.19]**	**0.000**	**-0.13**	**[-0.18, -0.08]**	**0.000**
**fimbria**	**-2.46**	**[-3.67, -1.26]**	**0.000**	**-0.13**	**[-0.20, -0.07]**	**0.000**
**HATA**	**-0.77**	**[-1.28, -0.26]**	**0.003**	**-0.09**	**[-0.15, -0.03]**	**0.005**
Sex						
Subfield	beta	95% CI	p	Std.beta	95% CI	FDR-p
**Hippocampal_tail**	**-31.82**	**[-47.74, -15.91]**	**0.000**	**-0.49**	**[-0.73, -0.24]**	**0.000**
**subiculum**	**-12.76**	**[-23.81, -1.71]**	**0.024**	**-0.25**	**[-0.47, -0.03]**	**0.028**
**CA1**	**-49.18**	**[-65.54, -32.83]**	**0.000**	**-0.63**	**[-0.84, -0.42]**	**0.000**
hippocampal fissure	-3.48	[-8.79, 1.84]	0.199	-0.16	[-0.41, 0.08]	0.200
**presubiculum**	**-14.10**	**[-22.33, -5.88]**	**0.001**	**-0.39**	**[-0.61, -0.16]**	**0.001**
**parasubiculum**	**-3.47**	**[-6.02, -0.92]**	**0.008**	**-0.33**	**[-0.57, -0.09]**	**0.011**
**molecular layer**	**-19.90**	**[-31.09, -8.71]**	**0.001**	**-0.38**	**[-0.59, -0.17]**	**0.001**
**GC-ML-DG**	**-23.13**	**[-30.37, -15.88]**	**0.000**	**-0.66**	**[-0.87, -0.46]**	**0.000**
**CA2/3**	**-12.42**	**[-18.44, -6.40]**	**0.000**	**-0.48**	**[-0.71, -0.25]**	**0.000**
**CA4**	**-16.90**	**[-22.50, -11.31]**	**0.000**	**-0.62**	**[-0.82, -0.41]**	**0.000**
fimbria	-4.18	[-8.55, 0.18]	0.060	-0.22	[-0.46, 0.00]	0.066
**HATA**	**-3.53**	**[-5.51, -1.56]**	**0.000**	**-0.42**	**[-0.66, -0.19]**	**0.001**
Age*Sex						
Subfield	beta	95% CI	p	Std.beta	95% CI	FDR-p
Hippocampal_tail	3.58	[-0.50, 7.65]	0.087	0.05	[0.00, 0.12]	0.149
**subiculum**	**3.74**	**[0.84, 6.63]**	**0.012**	**0.07**	**[0.02, 0.13]**	0.101
**CA1**	**4.84**	**[0.25, 9.42]**	**0.040**	**0.06**	**[0.00, 0.12]**	0.142
hippocampal fissure	1.53	[-0.19, 3.24]	0.083	0.07	[0.00, 0.15]	0.149

© 2024 Cai et al. *JAMA Network Open.*

presubiculum	2.38	[-0.02, 4.78]	0.054	0.07	[0.00, 0.13]	0.142
parasubiculum	-0.04	[-0.76, 0.68]	0.903	0.00	[-0.07, 0.06]	0.903
molecular layer	2.47	[-2.35, 7.30]	0.316	0.05	[-0.04, 0.14]	0.344
GC-ML-DG	1.19	[-0.82, 3.21]	0.248	0.03	[-0.02, 0.09]	0.305
**CA2/3**	**2.31**	**[0.43, 4.19]**	**0.017**	**0.09**	**[0.02, 0.16]**	0.101
CA4	1.17	[-0.54, 2.88]	0.180	0.04	[-0.02, 0.10]	0.270
fimbria	1.47	[-0.05, 2.99]	0.059	0.08	[0.00, 0.16]	0.142
HATA	0.37	[-0.27, 1.01]	0.254	0.04	[-0.03, 0.12]	0.305

Values in bold indicate statistically significant results. Abbreviations: cornu ammonis (CA), the granule cell and molecular layer of the dentate gyrus (GC-ML-DG), hippocampus-amygdala transition area (HATA).

eFigure 2. Developmental Trajectory of the Hippocampal Microstructure During Adolescence. Abbreviations: fractional anisotropy (FA), axial diffusivity (AD), mean diffusivity (MD), radial diffusivity (RD), axial kurtosis (AK), mean kurtosis (MK), radial kurtosis (RK).

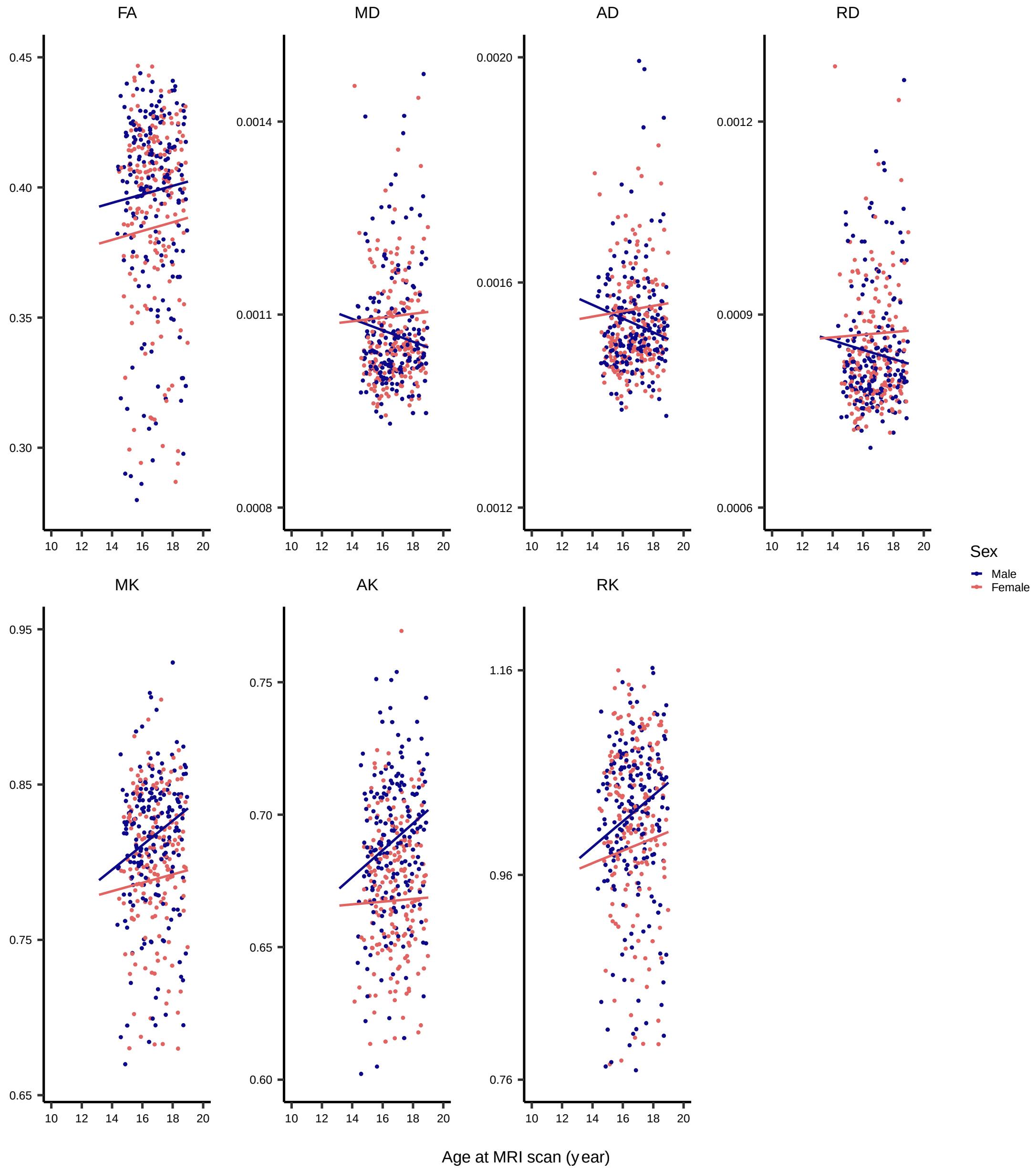

© 2024 Cai et al. *JAMA Network Open.*

eTable 2. Developmental Trajectory of Hippocampal Microstructure

Age						
Index	beta	95% CI	p	Std.beta	95% CI	FDR-p
FA	0.00	[0.00, 0.00]	0.437	0.05	[-0.08, 0.18]	0.437
AD	0.00	[0.00, 0.00]	0.059	-0.12	[-0.24, 0.00]	0.104
MD	0.00	[0.00, 0.00]	0.166	-0.09	[-0.21, 0.04]	0.232
RD	0.00	[0.00, 0.00]	0.278	-0.07	[-0.19, 0.06]	0.324
**AK**	**0.01**	**[0.00, 0.01]**	**0.001**	**0.20**	**[0.08, 0.32]**	**0.009**
**MK**	**0.01**	**[0.00, 0.02]**	**0.004**	**0.19**	**[0.06, 0.31]**	**0.013**
**RK**	**0.01**	**[0.00, 0.03]**	**0.009**	**0.17**	**[0.04, 0.30]**	**0.021**
Sex						
Index	beta	95% CI	p	Std.beta	95% CI	FDR-p
**FA**	**-0.01**	**[-0.02, 0.00]**	**0.011**	**-0.37**	**[-0.65, -0.09]**	**0.019**
AD	0.00	[0.00, 0.00]	0.168	0.20	[-0.08, 0.48]	0.168
MD	0.00	[0.00, 0.00]	0.107	0.23	[-0.05, 0.52]	0.125
RD	0.00	[0.00, 0.00]	0.093	0.24	[-0.04, 0.53]	0.125
**AK**	**-0.02**	**[-0.03, -0.01]**	**0.000**	**-0.75**	**[-1.03, -0.48]**	**0.000**
**MK**	**-0.03**	**[-0.04, -0.01]**	**0.000**	**-0.56**	**[-0.83, -0.28]**	**0.000**
**RK**	**-0.03**	**[-0.06, -0.01]**	**0.007**	**-0.38**	**[-0.65, -0.10]**	**0.016**
Age*Sex						
Index	beta	95% CI	p	Std.beta	95% CI	FDR-p
FA	0.00	[0.00, 0.00]	0.984	0.00	[-0.17, 0.18]	0.984
AD	0.00	[0.00, 0.00]	0.054	0.16	[0.00, 0.33]	0.189
MD	0.00	[0.00, 0.00]	0.177	0.12	[-0.05, 0.28]	0.309
RD	0.00	[0.00, 0.00]	0.308	0.09	[-0.08, 0.26]	0.380
**AK**	**-0.01**	**[-0.01, 0.00]**	**0.034**	**-0.18**	**[-0.34, -0.01]**	0.189
MK	-0.01	[-0.01, 0.00]	0.166	-0.12	[-0.30, 0.05]	0.309
RK	-0.01	[-0.02, 0.01]	0.326	-0.09	[-0.26, 0.09]	0.380

Values in bold indicate statistically significant results. Abbreviations: fractional anisotropy (FA), axial diffusivity (AD), mean diffusivity (MD), radial diffusivity (RD), axial kurtosis (AK), mean kurtosis (MK), radial kurtosis (RK).

© 2024 Cai et al. *JAMA Network Open.*

eAppendix 2. Supplementary Results 2: Sensitivity Analyses

To validate the main finding regarding changes in the mean hippocampal volume due to the SoE, we first used GAMM to reanalyze data with whole-wave missing values, among which only 58 participants had four repeated MRI measurements, and found a marginally significant main effect of SoE on the mean hippocampal volume (beta = 99.37, 95% CI [-6.64, 205.37], p = 0.07; Std. beta = 0.02, 95% CI [0.00, 0.05]). Second, we used the imputed data to conduct the same analysis and found no significant main effect of SoE on the mean hippocampal volume (beta = 108.41, 95% CI [-22.63, 239.45], p = 0.11; Std. beta = 0.03, 95% CI [-0.01, 0.06]).

The exploratory analysis aiming to examine the relationship between psychological distress and mean hippocampal volume showed no significant main effect of psychological distress on mean hippocampal volume (beta = -7.54, 95% CI [-36.26, 21.17], p = 0.59, Std. beta = -0.07, 95% CI [-0.32, 0.19]). Another two exploratory analyses also showed no significant main effect of the weekly number of days at school (beta = -61.43, 95% CI [-141.20, 18.35], p = 0.12, Std. beta = -0.21, 95% CI [-0.49, 0.06]) or the weekly extracurricular activity days (beta = -11.76, 95% CI [-63.83, 40.30], p = 0.642, Std. beta = -0.06, 95% CI [-0.30, 0.19]) on mean hippocampal volume.

© 2024 Cai et al. *JAMA Network Open.*

eTable 3. The Effect of SoE on Hippocampal Subfield Volumes

Main effect of SoE						
Subfield	Beta	95% CI	p	Std.beta	95% CI	FDR-p
Hippocampal_tail	-6.83	[-38.58, 24.91]	0.672	-0.01	[-0.07, 0.05]	0.897
subiculum	16.73	[-5.83, 39.29]	0.146	0.04	[-0.01, 0.10]	0.317
CA1	25.65	[-9.14, 60.44]	0.148	0.04	[-0.01, 0.10]	0.317
hippocampal fissure	-0.03	[-12.23, 12.17]	0.996	0.00	[-0.07, 0.07]	0.996
presubiculum	-0.31	[-18.24, 17.63]	0.973	0.00	[-0.06, 0.06]	0.996
parasubiculum	0.64	[-4.82, 6.10]	0.817	0.01	[-0.06, 0.07]	0.981
molecular layer	7.53	[-21.45, 36.51]	0.610	0.02	[-0.05, 0.09]	0.897
**GC-ML-DG**	**18.19**	**[2.97, 33.41]**	**0.020**	**0.06**	**[0.01, 0.12]**	0.118
CA2/3	8.54	[-4.91, 21.99]	0.213	0.04	[-0.02, 0.10]	0.365
**CA4**	**12.75**	**[0.38, 25.12]**	**0.044**	**0.06**	**[0.00, 0.11]**	0.174
fimbria	7.46	[-2.91, 17.83]	0.158	0.05	[-0.02, 0.12]	0.317
**HATA**	**5.67**	**[1.18, 10.17]**	**0.013**	**0.08**	**[0.02, 0.15]**	0.118

Values in bold indicate statistically significant results. Abbreviations: cornu ammonis (CA), the granule cell and molecular layer of the dentate gyrus (GC-ML-DG), hippocampus-amygdala transition area (HATA).

© 2024 Cai et al. *JAMA Network Open.*

eTable 4. The Effect of SoE on the Microstructure of the Hippocampus

Main effect of SoE						
Index	beta	95% CI	p	Std.beta	95% CI	FDR-p
**FA**	**0.03**	**[0.00, 0.06]**	**0.037**	**0.11**	**[0.00, 0.21]**	0.260
AD	0.00	[0.00, 0.00]	0.967	0.00	[-0.10, 0.10]	0.967
MD	0.00	[0.00, 0.00]	0.439	-0.04	[-0.14, 0.06]	0.614
RD	0.00	[0.00, 0.00]	0.275	-0.06	[-0.16, 0.04]	0.614
AK	0.00	[-0.02, 0.02]	0.918	0.00	[-0.09, 0.10]	0.967
MK	0.02	[-0.02, 0.06]	0.358	0.05	[-0.05, 0.15]	0.614
RK	0.05	[-0.02, 0.12]	0.143	0.07	[-0.02, 0.17]	0.501

Values in bold indicate statistically significant results. Abbreviations: fractional anisotropy (FA), axial diffusivity (AD), mean diffusivity (MD), radial diffusivity (RD), axial kurtosis (AK), mean kurtosis (MK), radial kurtosis (RK).

eAppendix 3. Supplementary Discussion

We also found increased volumes in the DG, CA4, and HATA subfields, which are in line with studies on both adolescents and adults demonstrating that stress is highly associated with volumetric changes in the hippocampal subfields.[19] For example, compared to trauma-exposed controls without PTSD, adults with PTSD had a decreased volume of the CA2–3, CA4, and DG.[20-22] In addition, compared to healthy controls, a significant volumetric reduction in HATA was found in PTSD adults with early childhood trauma.[23] A recent study found that the volume of CA2-3/DG region in both hemispheres was significantly smaller in adolescents with PTSD compared to healthy controls.[24]

We also found a trend increase of the microstructural integrity in the hippocampus. Although current literature regarding the effect of stress on the hippocampal microstructure is limited, a relevant study suggested that PTSD symptom severity had a negative correlation with hippocampal FA and a positive correlation with hippocampal MD.[25] A larger FA for adolescents assessed after the SoE shown in this study might indicate more pronounced tissue anisotropy, possibly resulting from increased axonal density, increased organization along the dominant axis, decreased interfering processes, and increased myelination.[26] However, we did not find any change in other DKI-derived indices. It may suggest that FA is more sensitive to the COVID-19 pandemic than other indices, which is in line with a PTSD study showing that FA is inclined to reach significance.[27]

© 2024 Cai et al. *JAMA Network Open.*

eAppendix 4. Supplementary Limitations

This study had several limitations. First, an exploratory analysis using psychological distress measurements showed no significant associations between stress level and hippocampal volume. Consequently, our study cannot provide a confirmative conclusion that structural changes in the hippocampus are only driven by stress. Given the limited biological and psychological stress measurements in this study, future studies are needed to investigate the relationship between the hippocampus and stress markers, such as cortisol level or psychological stress indices through a longitudinal approach. Moreover, to disentangle stress and the COVID-19 pandemic, long-term follow-up stress measurements for participants could offer insight into whether the hippocampal structures are more susceptible to individual stress events rather than the pandemic itself.

Second, changes in the environmental complexity during the SoE relative to those in other periods were not monitored in this study. For instance, participants were not asked to provide information on the time spent on learning, physical exercise, online classes, or doing homework. This limitation resulted in these confounding variables not being included in our statistical models. More well-planned behavioral measurements in future studies may help draw confirmative conclusions about the mechanism of action of a major global life event.

Third, our main significant findings were based on the complete case analysis strategy. If other strategies, such as multiple imputations were adopted to deal with missing waves for some participants, the results were not significant. However, at the current stage, the absence of data from approximately 150 participants in the fourth wave is attributed to ongoing data curation rather than participant loss. Additionally, it is important to note that the methodology of multiple imputation may not be appropriate for situations where data missingness exceeds 50%.[28] Consequently, the lack of significant results derived from multiple imputation may not be considered entirely convincing.

Fourth, since we changed the MRI acquisition procedure from wave 3, it allowed us to extract hippocampal subfield volumes and hippocampal microstructures. However, the non-linear regression approach could not be adopted due to the limited sample size. Because the effect size of the hippocampal volume was comparable to that of the subfield volumes and microstructural metrics, a smaller sample size and a narrower age range may result in non-significance after FDR correction.

Fifth, this study utilized automated hippocampal subfield segmentation based on FreeSurfer v6.0.0, primarily chosen for its suitability for large MRI scans. However, it is essential to consider its accuracy in comparison to manual segmentation. However, another popular automated segmentation tool, volBrain, is favored for its significantly shorter processing time. Moreover, a comparative study demonstrated a strong association in terms of hippocampal subfield volumes between these two segmentation tools.[29] Recently, more advanced methodologies, such as machine learning, may offer improved segmentation accuracy for investigating the structural plasticity of hippocampal subfields.[30]

© 2024 Cai et al. *JAMA Network Open.*

Finally, the adolescents in the pn-TTC are all residents of Tokyo, Japan. Notably, their parents possess higher levels of education. Consequently, caution is warranted when attempting to generalize these findings directly to adolescents residing in rural areas or those from low SES families in urban settings. Additionally, it is important to consider that hippocampal changes observed in the Asian population may not be universally applicable to other diverse racial populations.

© 2024 Cai et al. *JAMA Network Open.*

eReferences

1. Kessler RC, Andrews G, Colpe LJ, et al. Short screening scales to monitor population prevalences and trends in non-specific psychological distress. *Psychological medicine.* 2002;32(6):959-976.
2. Furukawa TA, Kawakami N, Saitoh M, et al. The performance of the Japanese version of the K6 and K10 in the World Mental Health Survey Japan. *International journal of methods in psychiatric research.* 2008;17(3):152-158.
3. Somerville LH, Bookheimer SY, Buckner RL, et al. The Lifespan Human Connectome Project in Development: A large-scale study of brain connectivity development in 5–21 year olds. *Neuroimage.* 2018;183:456-468.
4. Harms MP, Somerville LH, Ances BM, et al. Extending the Human Connectome Project across ages: Imaging protocols for the Lifespan Development and Aging projects. *Neuroimage.* 2018;183:972-984.
5. Yamashita A, Yahata N, Itahashi T, et al. Harmonization of resting-state functional MRI data across multiple imaging sites via the separation of site differences into sampling bias and measurement bias. PLoS biology. 2019;17(4):e3000042.
6. Maikusa N, Zhu Y, Uematsu A, et al. Comparison of traveling‐subject and ComBat harmonization methods for assessing structural brain characteristics. Hum Brain Mapp. 2021;42(16):5278-5287.
7. Glasser MF, Sotiropoulos SN, Wilson JA, et al. The minimal preprocessing pipelines for the Human Connectome Project. Neuroimage. 2013;80:105-124.
8. Wierenga LM, Bos MG, van Rossenberg F, Crone EA. Sex effects on development of brain structure and executive functions: greater variance than mean effects. J Cognitive Neurosci. 2019;31(5):730-753.
9. Herting MM, Johnson C, Mills KL, et al. Development of subcortical volumes across adolescence in males and females: A multisample study of longitudinal changes. Neuroimage. 2018;172:194-205.
10. Iglesias JE, Augustinack JC, Nguyen K, et al. A computational atlas of the hippocampal formation using ex vivo, ultra-high resolution MRI: application to adaptive segmentation of in vivo MRI. Neuroimage. 2015;115:117-137.
11. Sotiropoulos SN, Jbabdi S, Xu J, et al. Advances in diffusion MRI acquisition and processing in the Human Connectome Project. Neuroimage. 2013;80:125-143.
12. Andersson JL, Skare S, Ashburner J. How to correct susceptibility distortions in spin-echo echo-planar images: application to diffusion tensor imaging. Neuroimage. 2003;20(2):870-888.
13. Andersson JL, Sotiropoulos SN. An integrated approach to correction for off-resonance effects and subject movement in diffusion MR imaging. Neuroimage. 2016;125:1063-1078.

© 2024 Cai et al. *JAMA Network Open.*

14. Jensen JH, Helpern JA, Ramani A, Lu H, Kaczynski K. Diffusional kurtosis imaging: the quantification of non‐gaussian water diffusion by means of magnetic resonance imaging. Magnetic Resonance in Medicine: An Official Journal of the International Society for Magnetic Resonance in Medicine. 2005;53(6):1432-1440.
15. Garyfallidis E, Brett M, Amirbekian B, et al. Dipy, a library for the analysis of diffusion MRI data. Frontiers in neuroinformatics. 2014;8:8.
16. Bastiani M, Cottaar M, Fitzgibbon SP, et al. Automated quality control for within and between studies diffusion MRI data using a non-parametric framework for movement and distortion correction. Neuroimage. 2019;184:801-812.
17. Xie M, Cai J, Liu Y, et al. Association between childhood trauma and white matter deficits in first-episode schizophrenia. Psychiat Res. 2023;323:115111.
18. Taraku B, Woods RP, Boucher M, et al. Changes in white matter microstructure following serial ketamine infusions in treatment resistant depression. Hum Brain Mapp. 2023;44(6):2395-2406.
19. Leuner B, Gould E. Structural plasticity and hippocampal function. Annual review of psychology. 2010;61:111-140.
20. Wang Z, Neylan TC, Mueller SG, et al. Magnetic resonance imaging of hippocampal subfields in posttraumatic stress disorder. Archives of general psychiatry. 2010;67(3):296-303.
21. Hayes JP, Hayes S, Miller DR, Lafleche G, Logue MW, Verfaellie M. Automated measurement of hippocampal subfields in PTSD: Evidence for smaller dentate gyrus volume. J Psychiatr Res. 2017;95:247-252.
22. Luo Y, Liu Y, Qin Y, et al. The atrophy and laterality of the hippocampal subfields in parents with or without posttraumatic stress disorder who lost their only child in China. Neurological Sciences. 2017;38(7):1241-1247.
23. Ahmed-Leitao F, Rosenstein D, Marx M, Young S, Korte K, Seedat S. Posttraumatic stress disorder, social anxiety disorder and childhood trauma: Differences in hippocampal subfield volume. Psychiatry Research: Neuroimaging. 2019;284:45-52.
24. Postel C, Viard A, André C, et al. Hippocampal subfields alterations in adolescents with post‐traumatic stress disorder. Hum Brain Mapp. 2019;40(4):1244-1252.
25. Berman Z, Assaf Y, Tarrasch R, Joel D. Macro-and microstructural gray matter alterations in sexually assaulted women. J Affect Disorders. 2020;262:196-204.
26. Zatorre RJ, Fields RD, Johansen-Berg H. Plasticity in gray and white: neuroimaging changes in brain structure during learning. Nat Neurosci. 2012;15(4):528-536.
27. McCunn P, Richardson JD, Jetly R, Dunkley B. Diffusion tensor imaging reveals white matter differences in military personnel exposed to trauma with and without post-traumatic stress disorder. Psychiat Res. 2021;298:113797.

© 2024 Cai et al. *JAMA Network Open.*

28. Heymans MW, Twisk JW. Handling missing data in clinical research. Journal of clinical epidemiology. 2022;151:185-188.29. Samara A, Raji C, Li Z, Hershey T. Comparison of Hippocampal Subfield Segmentation Agreement between 2 Automated Protocols across the Adult Life Span. Am J Neuroradiol. 2021;42(10):1783-1789.

30. Singh MK, Singh KK. A review of publicly available automatic brain segmentation methodologies, machine learning models, recent advancements, and their comparison. Annals of Neurosciences. 2021;28(1-2):82-93.

© 2024 Cai et al. *JAMA Network Open.*
