# Supplementary material for: Hippocampal Structures Among Japanese Adolescents Before and After the COVID-19 Pandemic
Source: JAMA Netw Open. 2024 Feb 8;7(2):e2355292. doi: 10.1001/jamanetworkopen.2023.55292 (PMC10853829; doi:10.1001/jamanetworkopen.2023.55292)
Supplement: Supplement 2. — Data Sharing Statement [file jamanetwopen-e2355292-s002.pdf]

Data Sharing Statement

Cai. Hippocampal Structures Among Japanese Adolescents Before and After the COVID-19 Pandemic. *JAMA Netw Open*. Published February 08, 2024.
doi:10.1001/jamanetworkopen.2023.55292

Data
Data available: No
